# The Canadian pork industry and COVID‐19: A year of resilience

**DOI:** 10.1111/cjag.12276

**Published:** 2021-03-25

**Authors:** Ken McEwan, Lynn Marchand, Max Zongyuan Shang

**Affiliations:** ^1^ University of Guelph Ridgetown Campus Ridgetown Ontario Canada

**Keywords:** COVID, pandemic, pork, resilience, supply chain, COVID, pandémie, porc, chaîne d'approvisionnement, résilience

## Abstract

While COVID‐19 had the potential to be extremely disruptive to the Canadian pork supply chain, the sector showed resiliency by adjusting to market changes to ensure industry continuation. Unlike other non‐agricultural firms that were mandated to close at times, the pork sector was deemed an essential service and allowed to continue operating throughout the pandemic. Evidence of this resiliency is seen in three main ways. First, market access to the United States was maintained for both live pigs and pork exports. Second, Canada not only maintained market share in global pork exports, but it also actually increased shipments because of strong demand from China caused by African swine fever. Third, the challenges of processing plant closures and labour shortages were overcome in a variety of ways including increasing interprovincial shipments and increasing live pig exports to the United States. Pork consumption on a per capita basis continued the historical downward trend, and it is expected that consumers will return to their normal consumption patterns (e.g., dining at restaurants) despite job losses. At the meat processing level, it is anticipated that there will be an acceleration in the process to automate.

## Résumé

Bien que le COVID‐19 ait le potentiel de perturber considérablement la chaîne d'approvisionnement du porc canadien, le secteur a fait preuve de résilience en s'adaptant aux changements du marché pour assurer la continuité de l'industrie. Contrairement à d'autres entreprises non agricoles qui ont reçu l'ordre de fermer, le secteur porcin a été considéré comme un service essentiel et a pu continuer à fonctionner tout au long de la pandémie. La preuve de cette résilience est perçue de trois manières principales. Premièrement, l'accès aux marchés des États‐Unis a été maintenu tant pour les porcs vivants que pour les exportations de porc. Deuxièmement, le Canada a non seulement maintenu sa part de marché dans les exportations mondiales de porc, mais il a également augmenté ses expéditions en raison de la forte demande de la Chine causée par la peste porcine africaine. Troisièmement, les défis liés aux fermetures d'usines de transformation et aux pénuries de main‐d'œuvre ont été surmontés de diverses manières, notamment en augmentant les expéditions interprovinciales et en augmentant les exportations de porcs vivants vers les États‐Unis. La consommation de porc par habitant a poursuivi sa tendance à la baisse historique et on s'attend à ce que les consommateurs reviennent à leurs habitudes de consommation normales (e.g., manger au restaurant) malgré les pertes d'emplois. Au niveau de la transformation de la viande, on s'attend à une accélération du processus d'automatisation.

## 1 INTRODUCTION

COVID‐19 continues to cause tremendous upheaval in the world in terms of human health, market uncertainty, higher levels of unemployment, and restriction on the movement of people. While many non‐agricultural firms were mandated to close at times, the pork supply chain in Canada was deemed an essential service and allowed to continue operating throughout the pandemic. In the United States, an executive order invoking the Defense Production Act was signed by the President which ordered meat processing plants to remain open (USDA, 2020a, 2020b, 2020c).

There is limited literature on the impact of COVID‐19 on the pork sector because it is recent and still unfolding. However, there are a few articles discussing the impacts on the Chinese pork sector. Yu et al. (2020) found that the pandemic may reduce local pork prices as the strict quarantine measures adopted by China could lead to less demand. Ni et al. (2020) documented producers’ low willingness to recover pig production after the pandemic, although this could also be due to the heavy loss from African swine fever (ASF). Wang et al. (2020) suggested that the disruption of the pork supply chain in China is short‐lived. The discussion in North America revolves around processing plant backlogs. Lusk et al. (2021) discussed the price difference at farm and wholesale levels on the US pork supply chain. Hayes et al. (2021) provided details on how the US pork industry responds to processing plant closures such as slowing animal growth, increasing stocking densities, and adding more facilities.

This article describes the resiliency and adjustments made within the Canadian pork sector because of COVID‐19. It is an update to McEwan et al. (2020), which correctly identified the critical challenges for the industry as keeping the Canada/US border open for trade, maintaining global exports, and labour shortages causing temporary processing plant closures. The focus of this paper is on the resilience of the pork supply chain to overcome these challenges.

While COVID‐19 had the potential to be extremely disruptive to the Canadian pork supply chain with respect to US market access, global pork exports, and labour shortages, the pork sector showed great resiliency by adjusting to virus driven conditions to ensure industry continuation. Weersink et al. (2021) found that there is no general agreement on the definition of what makes a supply chain resilient. We view resilience as how robust and flexible the economic system is when facing unexpected external shocks such as an international trade dispute, natural disaster, or a pandemic. The Canada/US border has been closed to non‐essential traffic since mid‐March 2020, but agricultural trade between the two countries did not fall (Weersink et al., 2021). Evidence of resiliency is shown in the effort to keep the Canada/US border open to both live animal and pork trade. Additionally, Canadian global pork export volumes were not only maintained but increased by 17.9%. Pork processing plants operated on weekends to minimize a backlog in market weight animals, and there was timely live animal movement between provinces to prevent undesired animal outcomes [i.e., euthanasia (Ballingall, 2021)] caused by temporary plant closures and low prices.

## 2 CANADIAN HOG SLAUGHTER

**FIGURE 1:**
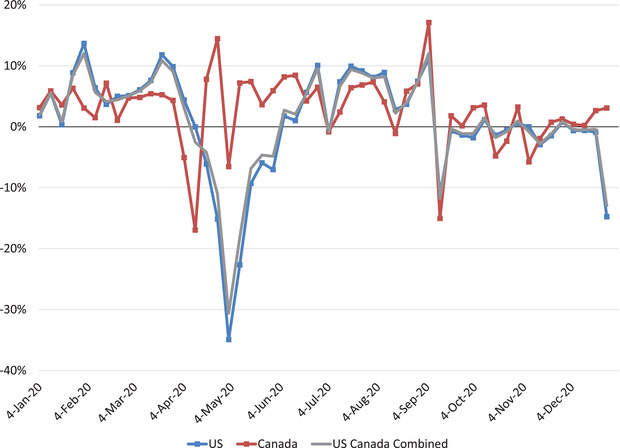
Weekly Percent Change in US and Canadian Hog Slaughter, 2020 Compared to 2019 *Data Source*: Calculations of percent change based on Agriculture and Agri‐Food Canada slaughter data and USDA, AMS SJ_LS711 report

In 2020, Canadian hog slaughter totalled 22.6 million heads, an increase of 4.0% over 2019, with provincial slaughter values of 8.4 million (Quebec), 5.9 million (Manitoba), and 4.7 million (Ontario) (Agriculture & Agri‐Food Canada, 2021). Interprovincial movement of pigs is common in Canada, but it increased in 2020 to respond to changes in processing plant capacities when COVID‐19 outbreaks occurred. For example, Ontario market hog volumes shipped to Manitoba and Alberta increased in 2020 compared to 2019 (i.e., an increase of 63,256 to Manitoba and 75,220 to Alberta) (Agriculture & Agri‐Food Canada, 2021). Personal conversations verified these shipments.^1^ The increase in total hog slaughter in 2020 combined with increased interprovincial movement of hogs helped manage animal numbers at the farm level. There were also changes in slaughter numbers within both Canada and the US during April and May 2020. Figure 1 shows the percent change in weekly slaughter numbers in 2020 compared to 2019 for the United States, Canada, and United States/Canada combined. Large declines from mid‐April to mid‐May signify the peak of US plant closures, but Canadian slaughter numbers did not exhibit the same impact. In fact, most Canadian plants were able to continue operating during this time and slaughter volumes increased to accommodate Canadian pigs that normally would have gone to US plants.

## 3 LIVE PIG EXPORTS TO THE UNITED STATES

The highly integrated nature of the Canadian and US pork industries allows for short‐term impacts of the pandemic to be seen by the changes in live animal trade flows. The majority of exports from Canada were 4.4 million feeder pigs contracted to US finishers and 802,871 market hogs for slaughter in 2020 (Canadian Pork Council, 2021b). Following the temporary drop in US slaughter capacity caused by the pandemic, US feeder pig prices declined sharply and the exports of Canadian feeder pigs fell by 21% year‐over‐year on average in May 2020.^2^ As US future market hog prices brightened and slaughter capacity became more certain, the demand for Canadian feeder pigs improved and shipments increased by 20.8% in September over May and June values.^3^ The integration of the Canada/US pork industry enables changes in animal flows depending on market conditions.

Further evidence of this integration is the export of an anticipated 80,000 to 90,000 market hogs to US packers due to the temporary closure of Olymel's Red Deer, Alberta processing plant in February 2021. Force majeure was declared due to an outbreak of COVID‐19, and the shipment of these hogs will help reduce the backlog of animals resulting from this closure (Nickel & Polansek, 2021).

## 4 CANADIAN PORK TRADE

While Canada has only 1.9% of the global sow inventory, it is the third largest pork exporter in the world followed by the EU (4.4 million metric tons) and the United States (3.3 million metric tons) (USDA, 2021b). In 2020, Canada exported 1.49 million metric tons of pork to 93 countries,^4^ which represented 73% of production (USDA, 2021b). Overall, Canadian pork exports in 2020 totalled $5.06 billion, which is an increase of 19.9% in dollar value and 17.9% in volume (kg) when compared to 2019 values. Exports to the United States were valued at $1.26 billion in 2020, similar to 2019. Interestingly, imports of US pork into Canada for 2020 were valued also at $1.26 billion and the US represented 86% of Canada's total pork imports.^5^

**FIGURE 2:**
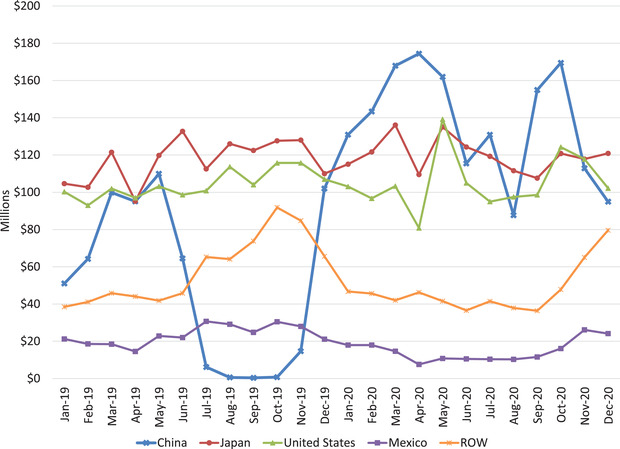
Monthly Canadian Pork Exports by Country in 2019 and 2020 *Data Source*: Statistics Canada, prepared by AAFC/MISB/AID/Market Information Section Note: The decrease in exports to China in 2019 was a result of a Chinese ban on Canadian pork from late June to early November 2019. The ban was a result of the potential finding of Ractopamine in a shipment of Canadian pork. Ractopamine is banned in China

China was the top export market for Canadian pork in 2020 reaching a value of $1.6 billion, which is an increase of 170% compared to 2019 and by 139% in terms of volume.^6^ Exports to China were strong to compensate for smaller domestic pork supplies resulting from ASF (see Figure 2).^7^ It is anticipated that China's pork imports may decrease somewhat from 2020 levels but will still remain strong over the next several months (Beek, 2021; USDA, 2021a, 2021d).

Despite the increase in pork exports from 2019 to 2020, there were challenges caused by COVID‐19. For example, there was a concern whether the virus that causes COVID‐19 could remain viable in food products or on food packaging. As a result, China indicated it would ban imports of meat from plants experiencing COVID‐19 outbreaks. The reasons given for the export suspensions were logistical issues and the potential for consumer backlash. China placed temporary suspensions on several Canadian pork exporters related to COVID‐19 reports at their facilities. However, the Maple Leaf Foods Brandon, Manitoba plant voluntarily suspended pork exports to China on August 19, 2020 after an outbreak was declared (Hirtzer, 2021).

**FIGURE 3:**
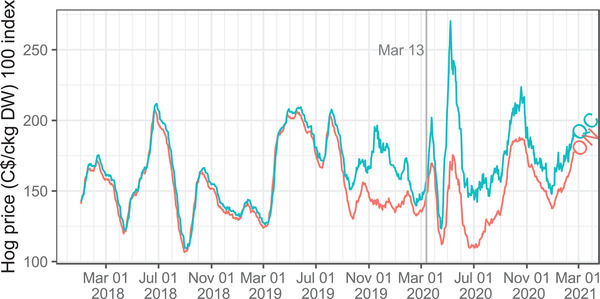
Daily Hog Prices in Ontario and Quebec *Data Source*: Ontario Pork (2021)

Hog prices at the farm level were volatile during 2020, particularly after March 13 (see Figure 3). The figure shows the unusually low prices in the summer of 2020 and it depicts, for Ontario especially, that producers endured several weeks of low prices. Quebec prices, which are tied to the USDA wholesale cutout price, were higher than Ontario but still very volatile. In Canada, government support was available to eligible hog producers that owned market‐ready hogs that had to be held back from shipping because of a temporary processing plant closure due to COVID‐19 outbreaks (Ontario Pork, 2020; RealAgriculture, 2021).^8^ In the United States, pig producers received payments through the Coronavirus Food Assistance Program (i.e., CFAP).^9^

**FIGURE 4:**
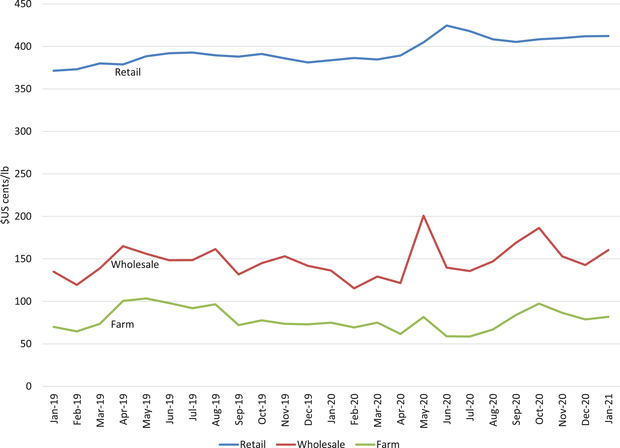
Average monthly US retail, wholesale and farm price *Source*: USDA (2021c)

During the first three months of the pandemic, the Quebec hog index price fluctuated between a wide range from $123/ckg DW to $270/ckg DW, a 120% increase from bottom to top (see Figure 3). While hog producers were facing price volatility, it is important to investigate what happened to prices at the wholesale and retail levels. Data from USDA (2021c) is used as an indicator because Canadian wholesale price data is not available. Figure 4 shows average monthly US farm, wholesale, and retail prices. While farm and retail prices remained fairly flat month to month, wholesale prices increased 65% in May 2020 compared to April 2020 (see Figure 4).

It is expected that Canadian processors also experienced higher wholesale prices, particularly in May as US plants closed in late April while most Canadian plants remained open. This may have contributed to the 32% increase in year‐over‐year pork exports to the United States in May 2020 as Canadian processors tried to benefit from the lower US supply of pork.^10^

## 5 LABOUR

McEwan et al. (2020) indicated that the pandemic could amplify the labour shortage in the pork industry through illness of current employees. It was acknowledged that throughout the value chain labour was critical but the potential closure of meat processing plants due to absenteeism was identified as being especially disruptive. In fact, at the time of publication of McEwan et al. (2020), several US and some Canadian pork processing plants experienced COVID‐19 outbreaks in their plants and they temporarily closed or reduced throughput. Cases at individual plants contributed to absenteeism by healthy workers due to fears of contracting the virus (i.e., SARS‐CoV‐2). In late May 2020, employee absenteeism at US plants was estimated to be as high as 50% out of fear of catching the virus that causes COVID‐19 (Polansek & Sullivan, 2020).

Plants implemented several policies in an attempt to minimize the number of cases. Some of these initiatives included installing physical barriers and distancing between employees when possible, employee screening, providing personal protective equipment, hand hygiene stations, and staggered start and break times. These measures appear to have significantly reduced outbreaks but came at a cost to processors.

A year after the pandemic started in North America, there are still occasional outbreaks at plants with some resulting in temporary shutdowns. This indicates that there remain vulnerabilities with respect to labour in the pork industry. As an example, because the plants are located away from city center and public transportation routes, in both Canada and the United States there are meat processing employees who sometimes carpool to work and this increases the potential for transmission (CDC, 2021; Groleau, 2020). Continued adherence to COVID‐19 public health guidelines is critical until vaccination of plant employees is complete.

## 6 CONCLUSIONS

To conclude, the Canadian swine sector displayed flexibility, resiliency and adjusted to the critical challenges posed by COVID‐19 to ensure industry continuation. The supply chain shared information between stakeholders to adjust to provincial, national, and international marketing challenges caused by the pandemic in order to meet shifting market demand. This resiliency was exhibited by keeping the Canada/US border open for trade, maintaining global market shares, and overcoming labour challenges. However, there were some difficulties experienced in the pork supply chain that can be directly attributed to the challenges arising from COVID‐19. These adversities that had to be overcome include lower producer prices, labour shortages causing temporary processing plant closures and strict export protocols from China. Still, the Canadian supply chain adapted quickly and worked together to prevent animal flow bottlenecks by moving pigs across Canada and to the United States.

The question remains as to what the new “normal” will be once businesses, including full‐service restaurants, return. By then the volume and demand for meat^11^ may be altered permanently or at least become slow to adjust to income effects associated with job losses.^12^ In addition, it is expected the process of automation will likely accelerate throughout the supply chain but mainly at the processor level. The implementation of labour saving technology (e.g., meat sorters, offal removers, loin removers, etc.) will be spurred by the costs associated with having to slow down line speeds, shut individual plants, have longer breaks between line shifts, increased cleaning costs, and lost market opportunities. Robotics that use central control technology (i.e., cameras, flow monitors, etc.) offer the greatest potential to limit virus spread among employees while maintaining plant efficiencies and ensuring a consistent, safe product.

Further, despite public interest in smaller processing plants to reduce employee concentration in one location and lower COVID‐19 transmission, this approach seems problematic given Canada's heavy dependency on foreign markets and the need for the pork to be federally inspected. North American processing plants are driven by economies of scale and typically run at line speeds of 1,000 hogs per hour or more with several operating 16 hours per day. Also, it is unclear how small provincial plants would reduce the transmission of the virus that causes COVID‐19 since workers would still be in close proximity to each other even though there are fewer of them. Still, reinvesting in provincial plants to supply local demand does seem reasonable. The historical imbalance between Canadian pig production and slaughter capacity continues but it is unlikely to be resolved with many small provincial processing plants.

## Footnotes

1 Personal conversations with industry specialists, producers, and provincial producer organizations.

2 Statistics Canada, Prepared by AAFC/MISB/AID/Market Information Section.

3 Statistics Canada, Prepared by AAFC/MISB/AID/Market Information Section.

4 Statistics Canada, Prepared by AAFC/MISB/AID/Market Information Section.

5 Statistics Canada, Prepared by AAFC/MISB/AID/Market Information Section.

6 Statistics Canada, Prepared by AAFC/MISB/AID/Market Information Section.

7 African swine fever is not currently in Canada but it is a robust virus with a high pig mortality (i.e., death loss from ASF can reach 100%). China reported its first case of ASF in August 2018. For 2020, USDA data forecasts indicate that China's pork production will fall more than 33% from 2018. Currently, the disease is in Africa, Asia, and parts of Europe (Canadian Pork Council, 2021a; Haley & Gale, 2020; Statistics Canada, 2020).

8 Depending on reference margins, Canadian producers may be eligible for program payments under the Business Risk Management suite of programs. Producer numbers remain stable at this time.

9 CFAP1 took into account pig sales between January 15, 2020 and April 15, 2020 and inventory between April 16, 2020 to May 14, 2020. CFAP2 payments were made on pig inventory numbers between April 16 and August 31, 2020 (USDA, 2020b, 2020a).

10 Statistics Canada, prepared by AAFC/MISB/AID/Market Information Section.

11 Pork consumption in Canada was estimated at 24.5 kg of carcass weight per capita in 2020 which is a 2.8% decrease from 2019. Canadian pork consumption has been stagnant to exhibiting a downward trend for several years and to illustrate this decline, in 2000 per capita consumption was 28.7 kg. As expected, COVID‐19 has had limited impact on overall pork consumption levels but has shifted demand away from foodservice and created increased interest in buying local (Burak, 2020; Statistics Canada, 2021b).

12 As of January 2021, the unemployment rate in Canada is 9.4%, the same as in August 2020 (Statistics Canada, 2021a).
